# The Telomere Length Signature in Leukemias—From Molecular Mechanisms Underlying Telomere Shortening to Immunotherapeutic Options Against Telomerase

**DOI:** 10.3390/cancers17121936

**Published:** 2025-06-10

**Authors:** Stella Baliou, Iordanis Pelagiadis, Miruna-Maria Apetroaei, Elena Vakonaki, Andreea Letiția Arsene, Eleftheria Hatzidaki, Manolis N. Tzatzarakis, Petros Ioannou, Aristides Tsatsakis, Eftichia Stiakaki

**Affiliations:** 1 Laboratory of Toxicology, School of Medicine, University of Crete, 71003 Heraklion, Greece; 2 Department of Pediatric Hematology-Oncology, University Hospital of Heraklion, 71110 Heraklion, Greece; 3 Laboratory of Blood Diseases and Childhood Cancer Biology, School of Medicine, University of Crete, 71003 Heraklion, Greece; 4 Faculty of Pharmacy, Carol Davila University of Medicine and Pharmacy, 6 Traian Vuia Street, 020956 Bucharest, Romania; 5 Marius Nasta Institute of Pneumophthisiology, 90, Viilor Street, 050159 Bucharest, Romania; 6 Department of Neonatology/NICU, University Hospital of Heraklion, 71110 Heraklion, Greece; 7 School of Medicine, University of Crete, 71003 Heraklion, Greece

**Keywords:** telomere length, leukemia, leukemia prognosis, oxidative stress, mitochondrial dysfunction, genomic instability, immunotherapy in leukemia, telomerase-based therapy, telomerase inhibitors, telomerase vaccines

## Abstract

The nucleoprotein structures known as telomeres provide genomic integrity by protecting the ends of chromosomes. Tumorigenesis is associated with alterations in telomere function and stability. This narrative review provides evidence of the potential prognostic value of telomere length and telomerase in leukemias. On the one hand, oxidative stress and mitochondrial dysfunction can accelerate telomere shortening, leading to higher susceptibility and the progression of leukemia. On the other hand, cytogenetic alterations (such as gene fusions and chromosomal abnormalities) and genomic complexity can result from checkpoint dysregulation, the induction of the DNA damage response (DDR), and defective repair signaling at telomeres. This review thoroughly outlines the ways by which telomere dysfunction can play a key role in the development and progression of four primary leukemias, including chronic lymphocytic leukemia (CLL), chronic myeloid leukemia (CML), and acute leukemias of myeloid or lymphoid origin, highlighting the potential prognostic value of telomere length in this field. However, telomerase, which is highly active in leukemias, can prevent the rate of telomere attrition. In line with this, leukemia cells can proliferate, suggesting telomerase as a promising therapeutic target in leukemias. For this reason, telomerase-based immunotherapy is analyzed in the fight against leukemias, leveraging the immune system to eliminate leukemia cells with uncontrolled proliferation.

## Simple Summary

Telomere shortening plays a significant role in the development of age-related diseases and cancer. However, this process can be reversed through telomerase, which is highly active in tumors. This narrative review examines the molecular mechanisms underlying increased telomere attrition, which contributes to an elevated risk of leukemias, highlighting the potential prognostic value of telomere length in leukemias. It emphasizes the connection between oxidative stress and mitochondrial dysfunction in accelerating telomere shortening, which contributes to a higher onset and progression of leukemia. Additionally, telomerase emerges as a therapeutic target in leukemias. For this reason, telomerase inhibitors and telomerase-based immunotherapy are analyzed in the fight against leukemias.

## 1. Introduction

Telomeres are repetitive nucleoprotein structures, in which an array of double-stranded TTAGGG repeats is assembled with shelterin complex proteins [1]. Telomeres consist of double-stranded telomeric repeats and the single-stranded G-rich 3′ terminal region (50–400 nucleotides), shielding chromosomes from fusion, destruction, and incorrect recombination [2].

The primary role of telomeres is to protect the ends of linear chromosomes to prevent the loss of coding regions and suppress large chromosomal rearrangements. Six telomere-associated proteins (TRF1, TRF2, POT1, TIN2, RAP1, and TPP1) comprise the shelterin protein complex, responsible for the structural arrangement of chromosome ends [3]. Shelterin protein components are believed to be essential for arranging telomeric ends into higher-order structures (T loops) to hinder the recognition of exposed DNA sites and the subsequent activation of DNA Damage Response (DDR) at telomeres [4]. From a structural perspective, a 3 G-rich overhang of telomeres is hidden in the formed T loop at the ends of chromosomes, preventing DNA damage and ensuring genomic stability [5].

During cell divisions, DNA polymerases cannot effectively replicate genomic portions at the ends of chromosomes, causing telomeres to be shortened [6]. The absence of telomerase sustains the gradual telomere shortening [6]. This telomere length reduction below a specific threshold fosters senescence and genomic instability [7].

When telomeres acquire a specific length, they cannot bind a sufficient number of telomere-binding proteins involved in the shelterin protein complex, and they become potentially exposed sites that induce DNA damage response and cell cycle arrest [8,9]. When telomeres become dysfunctional, the telomere structure disintegrates due to the relocation of the shelterin protein complex and the consequent DNA damage, resulting in senescence or apoptosis [8,9]. On the one hand, telomere-free ends can appear; they can be identified as double-strand breaks (DSBs) or single-strand breaks (SSBs), which trigger the overexpression of cell cycle inhibitors p16Ink4a, p21Cip1, and p53, that in turn induce senescence, which is then followed by apoptosis [10,11]. In the case of uncapped telomeres, they are associated with many chromosome abnormalities in the absence of cell cycle checkpoint mechanisms, thereby leading to genomic instability and cell death [12,13]. In particular, the inactivation or deletion of ataxia telangiectasia mutated (ATM)/ataxia telangiectasia and Rad3-related (ATR) or p53 transcription factor is associated with losing these repair mechanisms, which can trigger apoptosis or senescence. This loss of repair mechanisms is linked to end-to-end chromosomal fusions, fusion-bridge-breakage cycles, and overall genomic instability [14].

Telomere shortening is a common phenomenon closely linked to genomic instability and structural chromosome rearrangements due to increased breakage-fusion bridges [15]. Indeed, developing dicentric chromosomes can result from the dysfunction of the telomere nucleoprotein complex, exposing free chromosome ends to the DNA double-strand break (DSB) repair mechanism. Therefore, chromosomal instability can be induced by telomere loss or dysfunction, leading to cancer development and adverse clinical outcomes [16,17].

The incidence of hematologic cancers is rising as a result of a rapidly aging population, posing a challenge to health systems. Epidemiological transitions and demographic shifts have resulted in a heightened emphasis on malignant tumors globally [18]. Leukemia is a prevalent malignancy characterized by elevated blood leukocyte counts and the infiltration of bone marrow by blast cells. The precise etiology of leukemia has been inadequately comprehended so far. It is a complex illness arising from the interplay of genetic and environmental variables [19].

Leukemia, a potentially fatal condition caused by malignant clonal hematopoiesis of stem and progenitor cells, ranks among the top 10 diseases negatively impacting human health [20,21]. It leads to an uncontrolled proliferation of precursor cells of lymphoid or myeloid origin, resulting in bone marrow infiltration that impairs normal hematopoiesis. Unlike solid cancers, which often require genetic modifications and complex cellular reprogramming for effective metastasis, leukemic cells possess a unique, innate ability for migration and invasion. This ability is achieved by retaining the benign leukocytes’ capacity for cell motility and survival in circulation while simultaneously acquiring the potential for rapid and uncontrolled cell division [22]. Leukemia is classified into four main types based on the lineage affected and how quickly the disease progresses: acute lymphoblastic leukemia (ALL), more common in children, acute myeloid leukemia (AML), more common in older adults, chronic lymphocytic leukemia (CLL) which mostly affects adults over 60, and chronic myeloid leukemia (CML) which is less common, but the incidence increases with age [23].

Telomere shortening is regarded as the primary mechanism driving genome instability, which increases susceptibility to leukemogenesis and contributes to the progression of the four main types of leukemia. This association explains why telomere length is often considered a marker of poor prognosis in leukemia patients [24,25]. The clonal evolution of leukemia cells has also been linked to high telomerase activity. While a subgroup of cancer cells extend telomeres by telomerase-independent mechanisms known as alternative lengthening of telomeres (ALT) [26], telomerase has been understood to sustain telomere length in the overwhelming majority of cancer cells (80% to 90%) [27].

On the one hand, telomere biology disorders (TBD) are characterized by premature telomere shortening, thereby increasing the susceptibility to hematologic malignancies [28]. The major short-telomere syndromes are Dyskeratosis Congenita (DC), Hoyeraal–Hreidarsson syndrome, Revesz syndrome, and Coats Plus syndrome [29]. Apart from syndrome-specific characteristics, common clinical manifestations of short-telomere syndromes include idiopathic pulmonary fibrosis (IPF), aplastic anemia, hepatic fibrosis/cirrhosis, and myelodysplastic syndrome (MDS)/AML [28]. TBDs arise from mutations in genes involved in telomerase components (e.g., TERT, TERC), telomerase assembly and trafficking (e.g., NOP10, WRAP53), shelterin complex proteins (e.g., TINF2, POT1), or DNA repair and replication at telomeres (e.g., RTEL1, PARN) [30]. In contrast, long telomere syndromes emerge, wherein germline mutations affecting telomere-regulating genes, such as those responsible for protecting telomeres 1 (POT1), telomerase reverse transcriptase (TERT), telomerase RNA component (TERC), and regulator of telomere length 1 (RTEL1), can contribute to extended telomere lengths, thereby increasing the likelihood of developing cancer [31]. Interestingly, 70% of melanomas [32] and many other solid tumors [33] have somatic TERT promoter mutations. In this context, mutations in the following genes of the shelterin protein complex (POT1, TPP1, TERF2IP, and TINF2) have also been associated with familial melanoma, glioma, and chronic lymphocytic leukemia (CLL) [28].

Despite recent advancements, many questions about telomere biology and hematological cancers remain. Telomere attrition leads to genomic instability and disease development, particularly in leukemias. This comprehensive literature review discusses the molecular mechanisms that connect oxidative stress and mitochondrial dysfunction, resulting in telomere erosion and accelerating the onset and progression of leukemia. This review integrates molecular and clinical insights into telomere biology to enhance our understanding of the processes involved in leukemogenesis as well as the potential of telomere length as a potential prognostic marker for the onset and progression of leukemia. From a therapeutic perspective, telomerase is considered a promising therapeutic target for harnessing the immune system against leukemia cells.

## 2. The Molecular Mechanisms Underlying Telomere Shortening in Leukemias

Leukemia is a disease in which proliferation initially plays a significant role, resulting in the clonal expansion of abnormal myeloid or lymphoid cells [34]. Since leukemic cells proliferate quickly and evade typical cell cycle regulation, their telomeres are reduced with each cell division. This phenomenon is accentuated in specific leukemic clones with high turnover and insufficient telomerase activity. So, the short leukocyte telomere length can represent the higher cycling of the hematopoietic stem progenitor cell (HSPC) compartment or represent hereditary and acquired hematological dysfunctions, thus predicting the response to treatment in bone marrow failure syndromes (BMFS) and chronic myeloid leukemia (CML) [35].

Secondly, significantly reduced telomeric repeat sequences are another feature of many human malignancies that may lead to chromosomal arm telomeric fusions [36]. These fusions produce ring and dicentric chromosomes that, when cells divide, build bridges that can break and fuse broken ends to create new chromosome rearrangements.

Thirdly, dysfunctional telomerase can contribute to telomere attrition. In particular, people with impaired telomere maintenance bone marrow failure syndromes (BMFS) are characterized by telomere shortening and aberrant telomerase activity, which can lead to genome instability and organ failure [35,37]. Indeed, the inherited and acquired BMFS have been linked to either mutations in telomerase subunits but also to telomerase-binding proteins and members of the shelterin complex [35,37,38,39]. In this manner, unprotected telomeres are known to shrink, thus causing an activation of abnormal DNA repair pathways and chromosomal instability. Indeed, such genomic complexity can result from checkpoint dysregulation and the induction of DNA damage response and defective repair signals at deficient telomeres. In another case, elevated telomerase seemed to play a crucial role in driving chronic lymphocytic leukemia (CLL). In another example, androgenic anabolic steroids were shown to increase telomerase expression levels in the underlying hematopoietic stem progenitor cell (HSPC) compartment, thus sustaining telomere length values in leukocytes [40]. In line with this, a patient with aplastic anemia and a nonsynonymous mutation of the human telomerase reverse transcriptase (hTERT) gene sustained the telomere dynamics after long-term androgen treatment [40]. According to a number of studies, chronic lymphocytic leukemia (CLL) tumors exhibit significantly shorter telomere lengths but increased telomerase expression and activity compared to the values of normal B cells [41,42]. As a result, telomerase activation maintains the lowest possible telomere length necessary to evade senescence and maintain cell survival.

Fourth, a telomerase-independent mechanism, called alternative lengthening of telomeres (ALT), can sustain telomere length dynamics in leukemia cells [43]. Recent research has shown that ALT is activated in some subtypes of leukemia. ALT activation is often linked to mutations in chromatin remodeling genes including alpha thalassemia intellectual disability X-linked (ATRX) and death domain-associated protein (DAXX) [44]. In another study, 5% of the study participants had undetectable TERT expression and changes in ATRX- or DAXX, showing longer telomeres and higher telomeric repeat-containing RNA (TERRA) [45]. The activation of the ALT pathway can increase genetic instability. From this perspective, clonal evolution is facilitated, leading to the formation of leukemic subclones that are more resistant to therapy due to survival benefits. In this context, drugs that might efficiently target and disrupt the ALT pathway are currently under investigation to improve TKI efficiency for individuals with TKI-resistant leukemia. For instance, ponatinib has demonstrated synergistic effects in the eradication of ALT-positive cells when combined with other drugs such as triciribine [46].

Last but not least, epigenetic mechanisms play a crucial role in maintaining telomere length values in leukemias. The orchestration of the epigenetic landscape in telomeres can be accomplished through DNA methylation, histone modifications, and non-coding RNA like TERRA that together slowly impact hTERT expression or shelterin protein composition [47]. In this context, DNA methylation is one of several epigenetic changes that impact the accessibility of the TERT promoter to the transcriptional machinery, thereby affecting TERT expression in AML [48,49], For example, DNA Methyltransferase Inhibitors can increase the effectiveness of imetelstat, a telomerase inhibitor in high-risk AML patients [50].

### 2.1. The Oxidative Stress and Mitochondrial Dysfunction Loop Mediate Telomere Shortening

During homeostasis, mitochondrial respiration generates low reactive oxygen species (ROS) levels. In the mitochondria, adenosine triphosphate (ATP) is primarily produced to meet the metabolic demands of cells. Specifically, mitochondria synthesize ATP through oxidative phosphorylation (OXPHOS), in which the mitochondrial electron transport chain (ETC) complexes I–IV and ATP synthase (complex V) are essential. In the OXPHOS process, molecular oxygen is converted into water after electrons are transferred from complex I to complex IV of the mitochondrial ETC [51].

**Figure 1:**
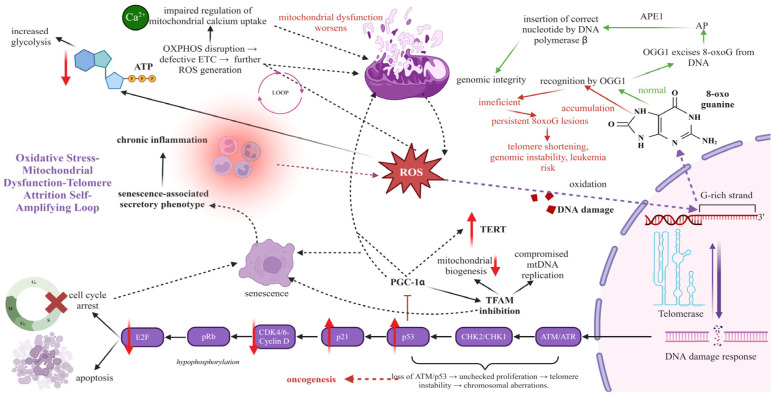
Oxidative stress–mitochondrial dysfunction–telomere attrition: A self-amplifying loop driving senescence and genomic instability. Reactive oxygen species (ROS) generated by defective oxidative phosphorylation (OXPHOS) impair the electron transport chain (ETC), further increasing ROS levels. ROS induces telomeric DNA oxidation, forming 8-oxo-guanine lesions, which disrupt the shelterin complex. This activates the DNA damage response (DDR) through ataxia telangiectasia mutated (ATM)/ataxia telangiectasia and Rad3-related (ATR) kinases, triggering checkpoint kinase 1 (CHK1)/checkpoint kinase 2 (CHK2), stabilizing p53, and upregulating p21, which inhibits cyclin-dependent kinase 4/6 (CDK4/6). This prevents retinoblastoma protein (Rb) phosphorylation, blocking E2F transcription factor activation and leading to cell cycle arrest, senescence, or apoptosis. Senescence-associated secretory phenotype (SASP) factors promote chronic inflammation, further amplifying oxidative stress. Mitochondrial dysfunction also suppresses peroxisome proliferator-activated receptor gamma coactivator 1-alpha/beta (PGC-1α/β), impairing mitochondrial biogenesis and energy metabolism. The loss of ATM or p53 function removes critical cell cycle checkpoints, leading to unchecked proliferation, chromosomal instability, and oncogenesis, reinforcing the loop (created with BioRender.com).

In most cases, ROS arise due to impaired oxidative phosphorylation at the mitochondria (Figure 1). On the one hand, the defects in the mitochondrial electron transport chain can drive ROS accumulation, exacerbating the oxidative stress that emerges during aging (Figure 1) [51]. On the other hand, the elevated ROS formation significantly disrupts the mitochondrial membrane potential, leading to decreased ATP synthesis and impaired energy metabolism, which further induces mitochondrial abnormalities (Figure 1) [51]. In this way, oxidative stress and mitochondrial dysfunction are closely intertwined.

In cases of mitochondrial dysfunction, the remarkable increase in mutations in mitochondrial DNA (mtDNA) causes the defective function of the electron transport chain (ETC) [52]. Indeed, mitochondrial ROS levels often reinforce these mitochondrial mutations in a positive feedback loop, exacerbating mitochondrial dysfunction [53,54]. Defective mitochondria are also prime candidates for oxidative damage, which impairs oxidative phosphorylation and increases reliance on glycolysis for energy generation [55]. Concurrently, mitochondrial calcium (Ca^2+^) accumulation significantly orchestrates mitochondrial metabolism and aging. The increased presence of senescent cells arises from reduced mitochondrial membrane potential due to elevated mitochondrial Ca^2+^ levels [56]. Senescent cells exhibit increased mitochondrial size, attributed to the heightened formation of malfunctioning mitochondria, leading to ROS accumulation [57].

The oxidative stress and mitochondrial dysfunction can contribute to genome instability through telomere shortening. A positive feedback loop has been highlighted regarding the relationship between oxidative stress, mitochondrial dysfunction, and telomeres. On one hand, mitochondrial damage mediates telomere dysfunction under oxidative stress conditions. Indeed, the mitochondrial ROS accumulation compromises telomere function, leading to the increased formation of telomere dysfunction-induced foci (TIF) and promoting senescence [58]. In particular, the mitochondrial damage causes p53 and DNA damage response (DDR) activation, eventually leading to telomere shortening (Figure 1). In line with this, TIFs occur when DNA-damage response (DDR) proteins are recruited to severely shortened and/or uncapped telomeres [59]. In another example, FCCP causes mitochondrial dysfunction through mitochondrial depolarization and ROS accumulation, leading to telomere shortening and chromosome fusions in mouse embryos [60]. In this context, it has been reported that the mitochondrial-targeted antioxidant Mito-Q can reduce telomere shortening and enhance the replicative lifespan of fibroblasts under mild oxidative stress by alleviating telomere shortening [61]. Consequently, mitochondrial dysfunction acts as a trigger for the amplification of telomere shortening. On the other hand, telomere disruption has been underlined to trigger a feed-forward loop composed of p53 activation and peroxisome proliferator-activated receptor gamma coactivator 1α/β (PGC1α/β) downregulation, thus impairing mitochondrial function and increasing oxidative defense (Figure 1). At the molecular level, the tumor suppressor gene p53 is activated, suppressing the peroxisome proliferator-activated receptor gamma coactivator 1a (PGC1α) and PGC-1β promoters, master regulators of mitochondrial metabolism (Figure 1) [57]. To support the above, the PGC1α induces mitochondrial biogenesis, handles mitochondrial dynamics, affects oxidative phosphorylation, and regulates mitochondrial genome replication. It is the key player in determining the amount of mitochondrial mass and their response to demands for energy [62].

### 2.2. The Activation of the DNA Damage Response Through Telomere Shortening and Mitochondrial Dysfunction

At the molecular setting, the telomere attrition causes impaired mitochondrial function through the activation of the DNA damage response [63]. In particular, telomere attrition stimulates DDR proteins, including H2A histone family member X (H2AX), eventually activating the tumor suppressor gene p53 [64]. Uncapped telomeres have been demonstrated to bind with DNA damage response factors, including 53BP1, MRE11, phosphorylated variants of H2AX, and ATM12, therefore initiating a signaling cascade that results in cell cycle arrest [65]. Consequently, telomere degradation activates H2AX, which in turn initiates p53. In reaction to DNA damage signals, p53 inhibits the production of SIRT1, PGC-1α, and PGC-1β (Figure 1). This inhibition ultimately results in a reduction of transcription factor A, mitochondrial (TFAM) levels, which is directly correlated with diminished mitochondrial biogenesis and compromised mtDNA replication (Figure 1). TFAM deficiency results in a diminished mtDNA copy number and a lower expression of mtDNA-encoded genes. TFAM-deficient cells exhibit elevated senescence-associated β-galactosidase activity and decreased cell growth characteristics [66].

### 2.3. The Association of Telomere Shortening with Metabolism

In addition, telomere dysfunction can affect energy metabolism since it represses PGC1α and SIRT1 deacetylase and activates p53 transcription factor. For example, telomere-dysfunctional mice have been shown to maintain normal levels of plasma glucose under fasting conditions due to deficiencies in gluconeogenesis controlled by the p53-mediated repression of PGC1a/b and its subsequent mediators, glucose-6-phosphate (GLC-6-P) and phosphoenolpyruvate carboxykinase (PEPCK) (Figure 1) [63]. The overexpression of mTERT or PGC1a, or genetic ablation of p53, led to an increased expression of PGC1α/b, GLC-6-P, and PEPCK, as well as the reactivation of gluconeogenesis [63]. Therefore, telomere dysfunction-induced mitochondrial impairment increases tissue reliance on glucose metabolism (Figure 1) [67].

To sum up, telomeres undergo a significant formation of 8-oxo guanine (8oxoG) defects under conditions of oxidative stress, since they are abundant in guanine [63]. In addition to this, mitochondrial dysfunction exacerbates oxidative defense, aggravating telomere shortening rate. As a result, accelerated aging arises from the positive feedforward loop between mitochondria, oxidative stress, and telomere dysfunction.

## 3. The Interplay Between Oxidative Stress, Telomere Length, and Leukemia Pathogenesis

### 3.1. The Key Role O of Oxidative Stress in Leukemias

Oxidative stress can promote carcinogenesis by altering the expression of genes linked to cancer. Chronic oxidative stress has been identified in certain leukemia patients [68]. The progression of leukemia can be affected by oncogenes that control the generation of ROS and the expression of antioxidants [69]. There is growing evidence that oxidative stress is a critical contributor to the development and prognosis of leukemia, and intriguing findings have been documented [70,71,72].

In AML, it has been proved that the prognosis of AML patients relies on the association of oxidative stress with signaling cascades and immune infiltration [20]. A key factor in the onset and progression of AML is the rise in oxidative stress levels [73]. Moreover, it has been reported that oxidative stress in AML patients can determine disease relapse [74]. For example, the pathobiological and recurrence processes of acute myeloid leukemia-M5 (AML-M5) are significantly influenced by ROS-mediated interactions between thioredoxin (TRX) and c-Jun activation domain-binding protein-1 (JAB1). Therefore, one possible therapy approach for AML-M5 could involve focusing on the ROS/JAB1/TRX pathway [75,76]. In this context, it has been supported that the oxidative nature of AML cells can guide the selection of therapeutic strategies to prevent relapse and drug resistance [77].

Regarding the relationship between oxidative stress and CML, it has been reported that CML is tightly linked to increased ROS recruitment. The CML disease is a common hematological malignancy that is caused by the uncontrollable enzymatic activity of a fusion protein known as Breakpoint Cluster Region-Abelson (BCR-ABL) [78]. This protein leads to granulocyte proliferation and immature differentiation in peripheral blood. CML cells can accumulate reactive oxygen species (ROS) due to the activation of NADPH oxidase and mitochondrial respiratory chain complex III (rac2/MRC-CIII) [79].

Regarding the association of oxidative stress and CLL, recent research suggests that oxidative burst can encourage the onset and progression of CLL [80]. While the antioxidant capacity level was significantly lower than that of the control group, the blood oxidation level was substantially higher in CLL patients [81], suggesting that the metabolic activity level of oxidative stress may be a good indicator of the stage at which the disease is progressing [82].

### 3.2. The Oxidative Stress Drives Telomere Shortening, Increasing the Susceptibility to Leukemias

Beyond the significant role of oxidative stress in leukemia progression, it is a key driver of telomere attrition, contributing to both aging and cancer development [83]. A negative association between telomere length and oxidative stress markers has been substantiated [84]. Convincing evidence suggests that stress causes respective cellular responses leading to telomere shortening. A meta-analysis supported a significant correlation between higher levels of perceived stress and lower telomere length [85]. A systematic review highlighted a negative association between different stimuli, including diseases, and telomere length [86]. In particular, oxidative stress may accelerate telomere shortening [87], driving senescence. A disequilibrium between the generation of reactive oxygen species (ROS) and cellular antioxidant defenses appears to be the main process responsible for the telomere dysfunction [88]. There is substantial evidence that oxidative stress accounts for the accumulation of DNA damage, thus potentiating telomere shortening and accelerating the incidence of age-related disorders [89].

Focused on the molecular mechanism underlying telomere shortening, mounting research has been conducted to shed light on this field. Initially, telomeric repeats are susceptible to oxidative damage since they are rich in guanine triplets, making them more vulnerable to oxidative DNA lesions than the rest of the genome [90]. It has been substantiated that oxidative stress accelerates telomere shortening, inducing DNA damage lesions. Up to 100 types of oxidized bases can arise from the effect of radicals on telomeres [91]. In telomeres, such lesions are single-strand breaks (SSBs), abasic sites, and fragmented pyrimidines and purines. Of all the bases, guanine is the most susceptible to oxidation. In particular, ROS accounts for the increased incidence of 8-oxo guanine (8oxoG) lesions in telomeres (Figure 1) [92]. Meanwhile, it has been noted that the 8oxoG lesions found in senescent cells are 35% greater than in normal cells [93] (Figure 1). Compared to genomic DNA, the adduct is present in this location in a more significant proportion—even seven times more [90].

Secondly, ROS have been shown to directly cause DNA damage by causing the increased formation of 8-oxo guanine (8oxoG) lesions in telomeres, oxidizing nucleoside bases [94], which, if left unresolved, can result in G-T or G-A transversions. The increased incorporation of 8oxodGTP opposite A at telomeres can generate TGAGGG repeats instead of GTAGGG and TGAGGG repeats during replication [94]. In line with this, 8oxodGTP has been demonstrated to operate as a blocking factor, interfering with telomerase activity and preventing telomere length extension mediated by telomerase. Telomerase enzyme does not fulfill its role when 8oxoG appears in the nucleotide pool as 8-oxodGTP [95,96]. As a result, these conversions can create DNA damage and lead to senescence due to the increased pace of telomere shortening [97].

Thirdly, oxidative stress can also accelerate telomere shortening, dissociating the binding of shelterin protein complex proteins at telomeres [98]. The six key proteins that constitute the shelterin complex—telomere repeat-binding factor 1 (TRF1), TRF2, protection of telomeres 1 (POT1), TPP1, TRF1-interacting nuclear factor 2 (TINF2), and RAP1—are essential in preventing telomeres from being identified as DNA damage-exposed sites. There is growing evidence linking the pathophysiology and development of leukemias to mutations in the expression of numerous shelterin components, especially POT1, TPP1, and TIN2 [42]. In CML, CLL, and AML, telomeres may become “uncapped”, or no longer shielded by the shelterin protein complex, when its function is compromised [25,99,100]. Furthermore, POT1 mutations are more prevalent in CLL types with poor prognosis, in which the patients exhibit severe clinical signs, increased telomere shortening rate, and complex cytogenetics [101]. On the molecular setting, telomeres become “uncapped”, triggering the ATR-associated DNA damage response [101]. In addition, TPP1 mutations are less commonly observed in leukemias; however, they have been detected in AML and myelodysplastic syndromes (MDS), which may impact telomere stability [37,100]. On the molecular setting, oxidative damage at telomeres may cause the displacement of shelterin proteins TRF1 and TRF2, potentially contributing to telomere dysfunction [98]. For example, ataxia telangiectasia and Rad3-related (ATR) kinases are activated when TRF2 has been eliminated from telomeres in oxidative stress conditions (Figure 1). In the case of TRF2 loss, the telomere dysfunction-induced foci (TIFs) are formed by the DDR activation, mediating phosphorylation signals to the downstream effectors checkpoint kinase 1 (CHK1)/checkpoint kinase 2 (CHK2) and stabilizing p53 transcription factor, thereby causing the accumulation of telomere dysfunction-induced foci (TIFs) (Figure 1) [102]. In this context, the DNA damage is manifested due to the histone variation H2AX and 53BP1 (p53-binding protein 1) (Figure 1) [103].

In dealing with oxidative DNA damage, 8-oxoguanosine DNA glycosylase-1 (OGG1) is an enzyme that can effectively remove the 8-oxoG insult from either nuclear or mitochondrial DNA [104]. When 8oxoG develops in opposition to C, OGG1 glycosylase identifies it, excising the lesion and forming an abasic site. Indeed, the ineffective OGG1 excision activity accounts for the inactivation of the base excision repair (BER) pathway, which can efficiently clear 8oxoG lesions from the D-loops and G-quadruplexes at telomeres (Figure 1) [104]. Meanwhile, the DNA polymerase fulfills its role, misincorporating A opposite 8oxoG. Then, MUTYH glycosylase removes A opposite 8-oxoG, generating a gap usually filled in by Pol λ or Pol β in the BER pathway [105]. As a result, the defective DNA repair systems stimulate telomere dysfunction by their inability to excise oxidized bases at telomeres (Figure 1) [104].

Oxidative stress also accelerates telomere shortening, since poly(ADP-ribose)-polymerase-1 (PARP1), the DNA damage sensor-dependent repair component of the base excision repair (BER) process, is ineffective [106]. Telomeric single-strand DNA breaks (SSBs) accumulate and eventually develop into potentially double-strand breaks (DSBs), which cannot be manipulated by the homologous recombination (HR) repair pathway or error-prone non-homologous end joining (NHEJ) [107,108].

In addition, the increased susceptibility of telomeric DNA base sequences to oxidative damage is also attributed to the more cumulative binding of iron (Fe^2+^) on telomeres than other sequences of the genome, thereby perpetuating the secretion of hydroxyl radicals through Fenton reactions [109]. Since the formation of oxidized bases is performed on average 100–500 times per day in the DNA of each cell in humans [110], 1200 oxidized bases in each cell cycle are expected [111].

Consistent with this, telomerase is considered critical for sustaining mitochondrial homeostasis because the TERT can protect mitochondrial function by reducing the levels of free radicals. The telomerase protects mitochondria due to its ability to increase the potential across the mitochondrial membrane [106]. According to studies, TERT can improve mitochondrial DNA repair and lower the formation of ROS in the mitochondria, both of which are essential for preserving cellular energy production and minimizing oxidative damage [112]. Maintaining cellular metabolism depends on TERT’s role in coordinating the expression of the mitochondrial genome. It binds to mitochondrial DNA (mtDNA), shielding it from oxidative damage. It also increases the production of electron transport chain components and antioxidant enzymes like superoxide dismutase, which improves mitochondrial function [113].

On the contrary, the oxidative stress accelerates telomere shortening by hindering telomerase action [95]. Telomerase activity has also been linked to oxidative stress. According to a molecular perspective, telomerase reverse transcriptase (TERT) is removed from the nucleus and translocated into the mitochondria due to an import leader sequence at the N-terminus of TERT when oxidative stress occurs [106,114,115]. As a result, the elimination of nuclear TERT inhibits telomerase action, preventing telomere shortening (Figure 1). However, mitochondrial telomerase fulfills its role of educating cells in oxidative stress conditions, leading to apoptotic cell death [106,115].

In this context, it has been reported that telomere dysfunction is related to genotoxic stress through the activation of the p53 transcription factor and to disturbed mitochondrial function through the downregulation of PGC1α and PGC1β gene expression [63]. Sahin et al. have highlighted the connection between telomeres and mitochondrial biogenesis in this context. Specifically, p53-mediated telomere damage disrupts mitochondrial respiration as the activated p53 tumor suppressor is recruited at the promoters of PGC1α and PGC1β, repressing the expression of genes involved in mitochondrial biogenesis [63]. As a result, oxidative stress, mitochondrial dysfunction, and telomere erosion are interconnected, resulting in genome instability and leukemia progression.

## 4. The Telomere Length Signature in Chronic Lymphocytic Leukemia (CLL)

**Figure 2:**
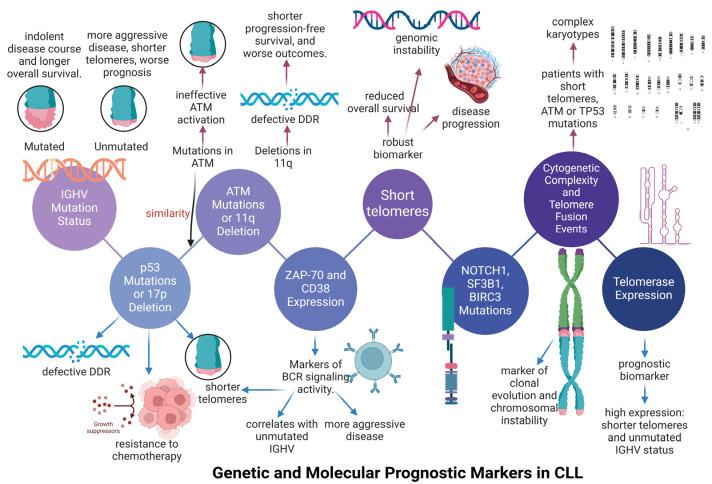
Genetic and molecular prognostic markers in Chronic Lymphocytic Leukemia (CLL). The immunoglobulin variable heavy-chain (IGHV) mutation status is a significant predictive factor, with mutated IGHV linked to indolent disease and unmutated IGHV associated with shorter telomeres and worse prognosis. Moreover, p53 mutations (17p deletion) and ataxia telangiectasia mutated (ATM) mutations (11q deletion) impair DNA damage response (DDR), leading to genomic instability, chemoresistance, and disease progression. Short telomeres could be biomarker of poor prognosis, driving chromosomal instability and clonal evolution. ZAP-70 and CD38 expression correlate with unmutated IGHV and aggressive disease, while NOTCH1, SF3B1, and BIRC3 mutations promote chromosomal instability and therapy resistance. Complex karyotypes and telomere fusion events are common in patients with ATM/p53 mutations, further worsening prognosis. Finally, high telomerase (hTERT) expression is linked to shorter telomeres, unmutated IGHV, and potential therapeutic targeting (created with BioRender.com).

Over the last fifteen years, there has been an increased focus on prognostic markers in chronic lymphocytic leukemia (CLL). Initially, genetic markers with high predictive value for CLL were recognized [116]. For example, the loss of the short arm of chromosome 17 (17p) or the loss of the long arm of chromosome 11 (11q), which includes the tumor suppressor gene p53 or ATM, constitutes the main prognostic genetic markers for CLL outcome. The mutation of the immunoglobulin heavy-chain variable (IGHV) region gene provides prognostic information regarding the aggressiveness of CLL disease [116]. Within this panel of predictive markers, the deficiencies in the following genes (BIRC3, NOTCH1, SF3B1) can yield valuable prognostic information (Figure 2) [116]. In addition to genetic changes, serum b2-microglobulin, clinical stage, and age, or Eastern Cooperative Oncology Group (ECOG) status, are also important prognostic factors (Figure 2) [116].

The unpredictable clinical trajectory of leukemias makes it essential to identify reliable prognostic markers that guide therapies and provide insights into outcomes. In this context, chromosome mutations, telomerase reactivation, and telomere shortening are recognized as risk factors for the emergence of leukemias [117,118]. In terms of CLL development, telomere length has two facets. On one side, elongated telomere length values may assist in tumor cell proliferation. On the other side, telomere shortening can also accelerate CLL progression, leading to genomic instability [117,118]. Several studies have provided convincing evidence that telomere length and telomerase expression have prognostic significance in CLL survival and response to therapy [41,119,120,121,122,123]. In most cases of CLL, telomere length is considered constant, but specific individuals who relapse have shown fluctuations in their telomere length values [42,124]. However, CLL patients with short telomeres have an unfavorable prognostic impact, often seen in those who relapse, displaying low overall survival (OS) [125] and a short time to first treatment (TTFT) [119]. Over the past decades, it has been established that short telomere length values are associated with advanced disease stages and lower OS, according to early research on the relationships between telomere length and survival in CLL patients [120,126]. Initially, Bechter et al. utilized telomere restriction fragment (TRF) analysis to demonstrate that short telomere length can be an adverse prognostic factor for CLL survival [120]. In particular, bone marrow samples with telomere length values less than six kilobase pairs have been shown to be linked to worse survival outcomes for CLL patients [120]. Similarly, Ricca et al. have shown that telomere length is a powerful predictor of time before starting treatment (TTFT), progression-free survival (PFS), and overall survival (OS), considering a threshold of telomeres to be 4250 base pairs, using TRF analysis [121]. Later, researchers reported that telomere length is an important predictor of time before starting treatment (TTFT) and survival for CLL patients [127]. In that study, low-risk patients seemed to avoid unnecessary therapy based on telomere length analysis [127]. Additionally, other researchers have demonstrated that telomere length could accurately identify patients with reduced survival and accelerated disease progression [123]. According to a recent meta-analysis, prolonged telomeres are linked to an increased overall survival of CLL patients, showing the prognostic significance of telomere length in CLL [128]. Later studies also revealed a positive relationship between short telomere length values and other detrimental characteristics of CLL pathology, such as lymphocyte doubling time [41] and the expression of CD38 and ZAP70 [125].

### 4.1. The Association Between Telomere Length and Immunoglobulin Variable Heavy-Chain (IGHV) Status in Chronic Lymphocytic Leukemia (CLL)

Extensive research has focused on the association between telomere length and immunoglobulin variable heavy-chain (IGHV) status, affecting the survival of CLL patients. When researchers conducted flow-FISH experiments, CLL patients with unmutated IGHV gene signatures presented shorter telomere length values than those with mutated IGHV [41]. The flow-FISH technique was conducted at polymorphonuclear leukocytes (PMNLs) or B cells isolated from the blood of B-CLL patients [41]. Similar results regarding the association of telomere length values with IGHV mutation status were observed when telomere length values were evaluated, using quantitative polymerase chain reaction (qPCR) [129]. Consistent with this, another study showed that the combination of telomere length with IGHV mutation status can provide information about the CLL outcome and survival [126]. Based on the above, the prognostic value of telomere shortening along with mutant IGHV status was highlighted [121]. Specifically, the researchers used TRF analysis to examine telomere length values, showing that telomeres can provide predictive information about progression-free survival (PFS), overall survival (OS), and time to first treatment (TTFT) of CLL patients [121]. Correspondingly, telomere length can provide predictive information about CLL progression and time to first treatment (TTFT) [119]. It was proved that CLL patients with IGHV unmutant status presented shorter telomeres, while an increase in the IGHV mutational burden was linked to longer telomeres of CLL patients [119]. Indeed, the prognosis for CLL patients with long telomeres and mutant IGVH is much better than that of patients with short telomeres and unmutated IGVH, suggesting that telomere length can be a potential marker for the IGHV mutant status [119]. In addition, another study has shown that telomere length can offer more prognostic information within the CLL groups with mutant IGHV status, being a better prognostic factor than IGHV status [129]. Lastly, it has been noted that CLL patients with unmutated IGHV present increased telomere shortening rates along with respective genomic aberrations [130].

In addition, researchers have employed TRF analysis to assess telomere length values in germinal center (GC) lymphocytes across a wide range of mature B-cell lymphoproliferative disorders (MBCLDs) [131]. It has been highlighted that telomere length is reduced in CLL disease and mantle cell lymphoma compared to diffuse large B-cell lymphoma, Burkitt lymphoma, and follicular lymphoma, suggesting that GC-derived tumors are associated with telomere elongation [131]. These findings indicate that GC-derived tumors have long telomeres and mutant IGHV status, since naïve B cells are characterized by unmutated IGHV status, and GC-mediated mature B cells exhibit mutated IGHV status [131]. Also, low proliferative neoplasias with short telomeres are shown to be treated more effectively using telomerase inhibitors [131]. However, other teams have demonstrated that patients with poor-prognosis, IGHV-unmutated, GC-inexperienced CLL cells have lower telomere length values than those from patients with IGHV-mutated CLL, clarifying the association between short telomeres and less favorable outcomes in CLL [41,126,129].

In addition, researchers have used the primary human tissues of CLL patients and proved the increased turnover of the neoplastic clone and subsequent short telomeres at cells can induce genomic instability, providing compelling evidence that CLL cells experience a telomere-based crisis that promotes CLL progression [132]. In particular, CLL cells had defective telomeres, using single-telomere length analysis (STELA) [133]. Additionally, the scientists pointed out that complete telomeric loss events, fusion events, and genomic rearrangements were centered at telomeres, implying that telomere shortening is a critical mediator of severe genomic instability [133]. In addition, patients with dysfunctional telomeres also had poor prognostic indicators of CLL disease including (Binet stage C, VH-unmutated status, and high Zap-70 expression) [133].

In order to elucidate the potential prognostic value of telomere length in CLL pathology, CLL patients under different therapeutic regimens were evaluated. Interestingly, high-risk, relapsed CLL patients treated with alemtuzumab were shown to have short telomeres that are significantly correlated with the limited progression-free survival (PFS) of CLL patients, suggesting that telomere shortening drives genomic instability in high-risk CLL patients [134]. Also, CLL patients treated with a chemoimmunotherapy (CIT) combination composed of fludarabine, cyclophosphamide, and rituximab (FCR) illustrated different survival outcomes depending on their genetic characteristics. In particular, IGHV-mutated patients presented significantly better overall survival (OS) than that of IGHV-unmutated patients, and patients with 17p deletion (p53 mutation) or 11q deletion (ATM mutation) experienced poorer outcomes with this regimen [99,135,136]. CIT resistance and decreased survival were shown to be linked to p53 alterations in CLL patients [137]. Low-burden p53 mutations were proved to exert a detrimental prognostic effect in CLL patients who did not receive treatment, since p53 mutations existed before the therapy of CLL patients and grew to become the predominant clone during patients’ relapse [137].

From this perspective, the potential prognostic value of telomere length in predicting the response to a FCR chemotherapeutic scheme in CLL patients was also underlined. Initially, CLL patients with short telomeres at the point where telomere fusions appeared showed worse overall survival than others [138]. In this way, the prognostic value of telomeres was proved, regardless of cytogenetic risk factors and IGHV mutation status [138]. The detection of telomere fusion events in early-stage patients with telomere shortening provided important predictive information about telomere length and CLL disease progression [133]. The presence of telomere fusions was shown to occur before CLL development, and their frequency increased with disease progression [133]. Indeed, the critical short telomeres start rounds of anaphase-bridging, breaking, and fusion, causing extensive genomic reorganization that could lead to the tumor’s clonal development and deficiencies in DNA damage checkpoint response [133]. In another study, researchers used high-throughput single telomere length analysis (STELA) assay to examine if CLL patients with telomeres inside the fusogenic range (TL-IFR) and CLL patients outside the fusogenic range presented differences in their overall survival (OS) and FCR response, taking into consideration that patients with very short telomeres at their chromosomes probably present chromosome fusion events [139]. It was proved that patients with telomeres inside the fusogenic range (TL-IFR) presented reduced FCR response, contributing to low overall survival (OS) and progression-free survival (PFS) [139].

### 4.2. The Association of Telomere Shortening with Genomic Abnormalities Occurred in Chronic Lymphocytic Leukemia (CLL)

Regarding the relationship between telomere length values and gene abnormalities, extensive research has been undertaken. In a molecular setting, short telomere length values in CLL patients have been proven to be associated with high-risk genomic abnormalities, including increased genomic complexity, 11q deletion/mutated ATM gene, and 17p deletion/mutated p53 gene, as well as the unmutated status of the immunoglobulin heavy-chain variable (IGHV) region gene. According to current research, patients with CLL who have short telomeres are significantly more likely to have deletions in 11q, 17p, and two or more cytogenetic abnormalities [138,140,141,142]. In particular, telomere shortening occurs in CLL patients when genomic areas (11q, 17p) are deleted. Following this, the activation of DNA damage response (DDR) occurs along with the upregulation of p53 and ATM critical checkpoint genes [130]. In leukemia, research has highlighted that short telomere length values provide a lot of selection pressure on CLL cells with unmutated IGHV to lose checkpoint genes like p53 or ATM, further leading to more pronounced telomere shortening and cell divisions [130,143]. These tumor cell clones with dysfunctional checkpoint genes (ATM or p53 deletion) accentuate the genome complexity with increased formation of breakage-fusion-bridge (BFB) cycles [125,130,144]

In addition, the relationship between telomere length values, chromosomal alterations, and karyotype has been extensively elucidated in CLL patients [140]. Initially, researchers used the quantitative polymerase chain reaction (qPCR) and quantitative fluorescence in situ hybridization (qFISH) experiments, and they demonstrated that telomere shortening is more pronounced in CLL patients with at least two aberrations (5). The qPCR analysis revealed that CLL patients had a significantly shorter mean telomere length than the control group [140]. Based on the FISH analysis and the cytogenetic characteristics of patients, it was noted that those with del11q/17p exhibited a lower median telomere length than CLL patients with the 13q14 deletion as a single alteration, in a statistically significant manner [140]. The median telomere length was higher in CLL patients with no aberrations and those with aberrant karyotypes compared to CLL patients with at least one aberration [140]. Following this, CLL patients with two or more alterations had a significantly lower treatment-free survival rate than those with no alteration (NA) and one abnormality [140]. Consequently, telomere shortening is shown to be crucial in promoting genomic instability in CLL, thereby substantiating the relationship between telomere shortening and chromosomal abnormalities in this disease.

In another study, researchers have elucidated the association between telomere length values and the function of the p53 and ATM genes in CLL patients [145]. More specifically, the researchers examined whether the complex karyotype in CLL patients is attributed to telomere shortening and loss of repair mechanisms [145]. To address this issue, the researchers used telomere/centromere fluorescence in situ hybridization (T/C-FISH), enhancing the ability to identify structural and numerical abnormalities in interphases and metaphases of chromosomes (translocations and dicentric chromosomes) after combining fluorescent in situ hybridization (FISH) in cytogenetic analysis [145]. The results proved that median telomere length was lower in CLL patients with complex karyotypes [145]. The accelerated telomere shortening rate was observed in CLL patients with mutant p53 status and complex karyotype [145]. Similar results arose to a lesser extent when the telomere length values were examined in CLL patients with complex karyotypes and ATM mutation [145]. As a result, a positive relationship between telomere length, specific cytogenetic aberrations, and genetic mutations was suggested [145]. In line with this, researchers employed a karyogram of CLL patients following the in situ hybridization of multicolor fluorescence (mFISH), which illustrated the complicated karyotype of CLL patients with unexpected abnormalities [145]. They showed that chromosome breakage points and the respective chromosomal translocations were more prevalent in nearby chromosome areas in CLL patients with aberrant karyotype and p53 deletion or mutation [145]. In contrast, patients with complex karyotypes without p53 involvement are characterized by less genome instability [145]. The above findings acquire more importance, taking into consideration that the T/C FISH labeling enables the accurate identification of chromosomal abnormalities, including the reciprocal translocation and the precise location of chromosome breakpoints [146].

In this context, another recent study has also reported that changes in telomere length values were associated with p53 mutation status and other related genomic features in CLL patients during their follow-up [147]. In particular, young CLL individuals exhibited a considerably higher frequency of short telomeres, based on qPCR analysis [147]. The increased telomere instability in young CLL individuals implied the evolution of leukemia clones with p53 mutations, suggesting a potential mechanism behind telomere shortening, which is independent of B-cell receptor (BCR) signaling [147]. This shows that telomere shortening and genomic p53 abnormalities in CLL patients provide poor prognostic information regarding CLL disease progression [147]. Indeed, a significant positive relationship was also observed between diminished BCR signaling activity and telomere shortening [147].

Interestingly, telomere dysfunction has been seen in CLL, which is associated with complicated karyotypes and chromothripsis. A series of detrimental genomic events begins to occur, including telomere crisis, which is defined by the critical telomere shortening and the general malfunctioning of telomeres. As observed in CLL, telomere shortening is associated with the increased generation of breakage-fusion-bridge (BFB) cycles in which chromosomes without telomeres fuse end-to-end and break during mitosis, thus increase genome instability [133,140,148,149]. Telomere crisis drives the positive selection of tumor clones with mutations that confer advantages for the proliferation of cancer cells, rendering them therapy-resistant. Interestingly, short telomeres and chromothripsis have been linked to reduced chemotherapy response in CLL [150].

### 4.3. The Inclusion of Telomere Length as a Parameter in Clinical Trial of Chronic Lymphocyic Leukemia (CLL)

The potential predictive significance of telomere length in CLL has been examined from a clinical perspective. In this context, a clinical trial has demonstrated that telomere length can play a crucial role in assessing the course of the illness. In particular, short telomere length values at diagnosis are associated with poor clinical outcomes, indicating the unfavorable prognostic significance of telomere length in CLL disease [124]. Notably, the positive association of telomere length with other adverse prognostic factors for CLL progression, such as p53, NOTCH1, and SF3B1 alterations, was reported [124]. In this clinical trial, telomere length remained stable in follow-up CLL patients 5–8 years after their diagnosis, regardless of the intervention [124].

Following this, another clinical trial showed that CLL patients with 17p- and 11q-associated p53 and ATM loss, respectively, exhibited the shortest telomeres, even when these abnormalities were present in minor subclones [42]. Thus, telomere shortening was observed to occur before the emergence of high-risk aberrations, contributing to the disease progression [42]. Consequently, telomere shortening is linked to both clonal evolution and an increase in genomic complexity, underscoring the significance of telomere length as a potential biomarker, especially in assessing the progression of non-high-risk CLL [42].

In line with this, a recent high-impact factor journal proved that only a tiny percentage of CLL patients achieved long-term progression-free survival (PFS). The prognostic markers that appeared to provide information for CLL patients following chemotherapy included the evaluation of p53 status, IGHV mutation status, telomere length, and CD49d expression [151]. The results of that study are consistent with the role of prognostic markers. Short telomeres, unmutated IGHV status, and increased CD49d expression in leukemia cells are linked to unrestricted cell proliferation, leading to greater genomic complexity. The p53 deficiency in leukemia cells seems to evade the apoptotic response to drugs, which is associated with chemotherapy resistance [151]. As a result, CLL patients with mutated IGHV, long telomeres, and absent CD49d expression following treatment with CIT showed a lower risk of relapse and improved PFS [151]. Notably, the inclusion of telomere length in the existing prognostic stratification algorithm makes it easier to identify a subset of patients who benefit most from FCR/FCR-based treatment plans [151].

In addition to the above, telomerase has been studied as a potential predictive biomarker for the progression of CLL. Importantly, researchers have claimed that human telomerase reverse transcriptase (hTERT) expression can have a potential predictive significance in B-CLL cases [127]. In particular, an inverse relationship was revealed between the percentage of IGVH mutational load and full-length (FL) transcript encoding the functional telomerase [127]. The overall survival of CLL patients with IGVH unmutated cases and low hTERT levels was comparable to that of CLL patients with IGHV mutant status and high hTERT levels, using the threshold of 40 copies of FL hTERT transcripts [127]. In this manner, the length of the hTERT transcript provides prognostic information about the overall survival of CLL patients [127].

To sum up, telomere length and telomerase expression can be potentially prognostic biomarkers in providing information about CLL survival, progression, and response to therapy.

## 5. The Telomere Length Signature in Chronic Myeloid Leukemia (CML)

The clonal, myeloproliferative disease known as chronic myeloid leukemia (CML) begins with aberrant changes in the hematopoietic stem and progenitor compartment (HSPC), thus leading to a significant increase in myeloid cell size [152]. In CML, the Philadelphia chromosome (Ph) and the BCR-ABL fusion oncogene are primarily generated when chromosome 9 and chromosome 22 undergo a reciprocal translocation (t(9;22)), accounting for unlimited cell proliferation and resistance to apoptosis [153]. In general, the clinical course of CML includes a relatively stable chronic phase (CP) that lasts for several years before moving into an accelerated phase (AP) and ultimately blast crisis (BC) [25,154]. In the CP of CML disease, less than 10% of the cells in the bone marrow and blood are blast cells in chronic phase CML [25,154]. In the AP of CML disease, blast cells constitute 10% to 19% of the blood and bone marrow. In the blast phase of CML disease, at least 20% of the bone marrow or blood cells are blast cells [25,154].

Initially, it was shown that the telomere length of Ph+ peripheral leukocytes was shorter by one kilobase compared to that of controls [155]. In line with this, fluorescence in situ hybridization and flow cytometry experiments proved that the telomeres of leukocytes derived from CML patients were significantly shorter than those of age-matched controls and normal lymphocytes from the same individual [156]. On the molecular setting, the increased telomere shortening in the peripheral blood myeloid cells in CML patients was substantiated by the accelerated cellular turnover of clonal BCR-ABL-positive hematopoietic stem progenitor cells (HSPCs) [156]. Compared to cells from CML patients in chronic phase (CP) or cytogenetic remission, leukocytes in accelerated phase or blast phase (AP/BP) displayed a noticeably more pronounced telomere shortening rate [156]. In particular, telomeres of leukocytes were noticeably shorter in patients who transitioned from CP to BP of CML disease within two years than in those who did not [156]. In addition, it was highlighted that telomere length reduction in the leukocytes of CML patients was substantially correlated with time from diagnosis to AP but not during the progression from diagnosis to BP [156]. Overall, telomere shortening in CML patients emerged as a strong prognostic marker for disease progression [156]. In this context, another study has reported that the telomere length in leukocytes of CML patients was reduced by 2.2 kilobases compared to that of controls [157].

Based on this, the telomere length in CML patients was studied at all phases of the disease, and the follow-up of CML patients was based on the cytogenetic response to imatinib mesylate [158]. Follow-up measurements were performed on patients who achieved either a complete cytogenetic response (CCR) or no complete remission (CR) [158]. The flow-FISH experiments showed that telomeres in the accelerated phase (AP) and blast crisis (BP) of CML patients were significantly shorter than in the chronic phase (CP) of CML patients. Importantly, the same study showed that the mean telomere shortening was substantially more significant in high-risk CML patients with high Hasford score than in low-risk patients at diagnosis [158]. As the CML disease progresses, the rate of shortening was observed to be 10–20 times observed to be increased than that of normal granulocytes [158]. In addition, the flow-FISH technique was applied to peripheral blood leukocytes in CML patients.

In CML, telomere length can be a prognostic marker for monitoring therapy effectiveness. According to ELN criteria, patients with the longest telomeres have a lower clinical risk profile than those with shorter telomeres, suggesting the importance of telomeres in predicting disease progression [159]. The telomere length values can provide essential insights into TKI responsiveness after 12 and 18 months [159]. Regarding tyrosine kinase inhibitor (TKI) treatment, individuals who had been taking imatinib for over 144 days exhibited noticeably longer telomeres than those who had just initiated TKI treatment [160]. In comparison to samples from patients with minor cytogenetic response or no cytogenetic response, telomere length was found to be greater in samples from patients in significant or complete cytogenetic remission [160].

Besides the potential prognostic value of telomere length, telomerase also has potential prognostic implications during CML disease progression. Telomerase seems to provide crucial predictive information about the CML disease progression and survival [155,161]. Interestingly, there is a correlation between telomerase activity and the development of acute vs. chronic types of CML [155,162]. Initially, it was shown that telomerase activity was significantly higher in the BP than in the CP of CML disease, considering that CP-CML showed detectable telomerase activity above baseline [155]. In particular, telomerase activity is increased in CP-CML and may rise by up to 50 times in the BP of CML patients [155,162].

From a therapeutic perspective, BCL-2 and Bruton’s tyrosine kinase inhibitors (BTKis) affect the telomere length dynamics in chronic myeloid leukemia (CML). According to a study, BTK has a crucial role in CML’s imatinib resistance [163]. The imatinib targets the fusion BCR:ABL1 gene that forms the so-called Philadelphia chromosome [163]. Nevertheless, there is currently little direct evidence connecting BTK inhibitors to alterations in telomere length in CML. Accordingly, telomerase activity may be decreased by BCL-2 inhibition, implying a reduction in telomere length values, considering that increased telomerase activity has been linked to BCL-2 upregulation [164]. Although venetoclax and other BCL-2 inhibitors are approved for use in several blood cancers [165,166], the effect of these inhibitors on telomere length values in CML remains unknown.

In addition, the treatment of chronic myeloid leukemia (CML), particularly in patients who are resistant or intolerant, has changed with the introduction of second-generation TKIs (such as dasatinib, nilotinib, and bosutinib) and third-generation TKIs (such as ponatinib and asciminib) [167]. Progressive telomere shortening is frequently seen in patients who achieve long-term molecular remission, especially in CD34+ progenitor cells [168].

Regarding the change in telomere length dynamics from used therapeutic in CML, a clinical trial examined whether the telomere length changes in peripheral blood granulocytes were related to therapy response with the selective tyrosine kinase inhibitor (imatinib) [169]. During the therapy response of CML patients up to 144 days, they had shorter telomeres than those of CML patients after 144 days of therapy response [169]. In another clinical trial, the degree of telomere destruction in Ph-negative hematopoiesis following a successful therapy for CML was assessed over a 22-month follow-up period [170]. The results showed that CML patients in complete cytogenetic remission had accelerated telomere shortening compared to healthy individuals [170]. In line with this, a recent clinical trial compared the telomere length of non-leukemic BCR ABL-CD34+CD38 negative hematopoietic stem cells (HSC) to that of their BCR-ABL positive leukemic stem cell (LSC) derived from the bone marrow of CML patients at diagnosis [168]. As anticipated, LSC exhibited substantially more significant telomere shortening than non-leukemic cells. Indeed, the degree of LSC telomere shortening is related to the number of leukemic clones, suggesting that telomere shortening is a predictable consequence of uncontrolled leukemia cell growth [168]. No statistically significant differences were observed in granulocytes between AML patients and controls. The results of this clinical trial provide compelling evidence regarding the prognostic value of telomere length in CML [168]. To sum up, the prognostic value of telomere length is proven in CML disease during pathogenesis and therapy response.

## 6. The Telomere Length Signature in Different Types of Acute Leukemias

Acute leukemia represents a disease caused by clonal expansion and arrest at a specific stage of myeloid or lymphoid hematopoiesis. Leukemia is a disease characterized by cytogenetic and molecular abnormalities, and it has significant biological and prognostic implications that impact treatment stratification. A large number of chromosomal rearrangements characterize both types of ALL [171]. The origin of ALL is immature T or T lymphoid progenitors, whereas myeloid progenitors give rise to AML. Known genetic alterations in B-ALL are hyperdiploidy or hypodiploidy, t(12;21) or ETV-RUNX1 fusion gene, t(9;22) or lysine-specific methyltransferase 2A (KMT2A) gene rearrangements, IKZF1 deletion or intrachromosomal amplification of chromosome 21 (iAMP21) or t(1;19) TCF3-PBX1 fusion gene. Each of the above cytogenetic abnormalities carry prognostic significance and aid in risk stratification and the therapeutic management of ALL patients [172]. Besides the TERT and the shelterin protein complex, expression varies according to type of ALL [173]. ALL therapy includes multi-agent chemotherapy with CNS prophylaxis and hematopoietic stem cell transplantation (HSCT) in high-risk or relapsed patients. Targeted therapies are added in cases of known targetable genetic lesions, such as TKIs in Philadelphia positive B-ALL [174,175]. The common genetic abnormalities of AML are the following: translocation between chromosomes 15 and 17 (PML-RARA in APL), translocation between chromosomes 8 and 21 (RUNX1-RUNX1T1), FLT3-ITD, NPM1, CCAAT Enhancer Binding Protein Alpha (CEBPA) mutations, the inversion of chromosome 16, and KMT2A rearrangement [176]. Telomere shortening is a characteristic feature of high-risk AML [96]. Most AML treatment protocols are based on chemotherapy with anthracyclines and cytarabine, with HSCT reserved for high-risk or relapsed patients. Currently used novel therapies include anti-CD33 monoclonal antibody, targeting inhibitors against Fms-like tyrosine kinase 3 (FLT3) and isocitrate dehydrogenase 1 and 2 (IDH1/2) and venetoclax, a BCL2 inhibitor. Acute promyelocytic leukemia (APL) is treated with all-trans retinoic acid (*atRA*) and arsenic trioxide (ATO) [177,178].

Acute Lymphoblastic Leukemia (ALL) accounts for 80% of all childhood leukemias, of which 85% are B-cell ALL (B-cell) and 15% T-cell (T-ALL) [179]. There are distinct molecular markers for T-ALL and B-ALL cells. Some of them, including cytokine receptors and protein kinases, are reliant on chromosome rearrangements and mutations in lymphocytes [172]. Remarkably, up to 75% of childhood acute lymphoblastic leukemia cases exhibit evidence of chromosomal gains, losses, or translocations.

Adult ALL has generally had a poor prognosis, with limited treatment options and a cure rate of less than 40% compared to pediatric ALL [180]. More than 70% of the various forms of leukemia that occur in children are caused by ALL [181]. Adult patients exhibit more cooperative mutations, fostering epigenetic changes that may lead to the formation of B cells [182]. In children, the overall five-year event-free survival rate for the disease exceeds 90%. However, recurrences result in the deaths of 10–20% of patients, which is highly fatal [183].

Regarding the prognostic markers of B-cell acute lymphoblastic leukemia (B-ALL), age and white blood cell count are two significant predictors of the outcomes in B-ALL [184]. Patients aged between 1 and 10 years with an initial white blood cell count (WBC) of less than 50,000/mm^3^ [standard risk (SR)], which encompasses two-thirds of B-ALL patients, have an event-free survival (EFS) of over 80% within 4 years. Conversely, the remaining patients [high risk(HR)] have an EFS of 75% within 4 years [184]. Regarding age, patients under 1 year of age and those over 10 years of age have a worse prognosis than children between 1 and 10 years of age. Infants under 1 year of age have the worst prognosis [184]. Regarding white cell count, children with a higher WBC tend to have an inferior prognosis. In addition, factors that are considered in risk classification include the immunophenotype and cytogenetics of ALL patients. Cytogenetics has revealed that combinations of chromosomes 4 and 10 trisomies are associated with favorable outcomes. Similarly, translocations involving ETV6-RUNX1 are also linked to excellent prognoses. However, translocations involving the MLL rearrangement on 11q23 are associated with inferior prognoses. Historically, the Philadelphia chromosome t(9;22) (q34;q11) ALL has been associated with a poor prognosis [184]. However, incorporating tyrosine kinase inhibitors (TKIs) into the intensive chemotherapy regimen has significantly improved the disease’s outcome. Hyperdiploidy, characterized by a chromosome number above 50 or a DNA index of 1.16, is associated with a favorable prognosis. On the contrary, patients with hypodiploid blasts, having fewer than 44 chromosomes or a DNA index of 0.81, have a significantly poorer prognosis [184].

Noteworthy, the presence of central nervous system (CNS) disease at diagnosis is an adverse prognostic factor despite the intensification of therapy with CNS irradiation and additional intrathecal therapy [184]. Of particular significance is the prognosis for patients who do not achieve remission at the conclusion of induction therapy. Individuals with positive Minimal Residual Disease (MRD) at the end of induction therapy exhibit a significantly unfavorable prognosis [184].

In general, acute leukemia patients are characterized by telomere shortening compared to controls [185]. Initially, Southern blot analysis proved that this decreasing trend of telomere length values is accelerated when acute leukemia patients relapse [185]. Furthermore, B-ALL patients have shorter telomere length values compared to those of T-ALL patients [185]. In contrast, a negative correlation between telomere length and telomerase has been observed in acute leukemia patients [185]. Compared to patients with early-stage disease, those with late-stage disease have higher telomerase activity and shorter telomere length values, presenting more adverse five-year survival [185]. Overall, the short telomere length values in combination with high telomerase activity are proven poor prognostic indicators of acute leukemia, contributing to advanced disease progression and relapse [185].

Regarding telomere length in ALL, T-ALL patients present with longer telomeres than B-ALL patients. Indeed, ALL patients with complex karyotypes exhibited a faster telomere shortening rate than all patients with normal karyotypes [186]. The increased rate of telomere shortening can also provide predictive information about the aggressiveness of ALL [186]. Recently, it has been underlined that the increased recovery rates of B-ALL are associated with low telomerase activity and telomere length elongation [173].

In the majority of studies, it has been shown that acute leukemias have high telomerase activity and short telomere length values. According to Capraro et al., telomere length is significantly lower in AL patients with an aberrant karyotype compared to those with a normal karyotype, and the shortest telomeres are found in individuals with multiple abnormalities. In particular, all cases with aberrant karyotypes had shorter telomeres than those of ALL patients with a normal karyotype, with telomerase activity being greater in B-ALL cases compared to those of T-ALL cases [186]. In line with this, male patients have been found to have a higher telomerase activity in ALL cells than female patients. This could be because estrogen has an adverse regulatory effect on telomerase [187].

In addition, it is essential to note that the levels of telomerase in adults’ lymphoblasts with ALL have been observed to be lower compared to those in patients with pediatric ALL [188,189]. In this manner, the telomeres in adults’ lymphoblasts are shorter than those of children. In lymphoblasts of high-risk pediatric patients at diagnosis, the telomerase levels are increased, contributing to the establishment of telomerase as a prognostic marker in children with ALL [188,189]

Even though allogeneic hematopoietic stem-cell transplantation (HSCT) offers children with high-risk ALL exceptional cure rates, relapses continue to be the primary cause of treatment failure [190]. The donor-killer cell immunoglobulin-like receptor (KIR) genotype has been shown to affect the recurrence rate in pediatric ALL after hematopoietic stem cell transplantation (HSCT). Specifically, the presence of KIR B haplotypes at the centromeric region and their absence at the telomeric region are associated with a decreased risk of relapse in children with ALL [190].

In acute leukemias, AML is a type of aggressive cancer arising from gene or chromosome mutation in blood or bone marrow cells and is more prevalent in adults aged 60 and older. Although at least one cytogenetically identifiable lesion is present in 55% of AML patients at diagnosis, the remaining 45% are not [191].

Several studies have elucidated the role of telomere length in different types of acute leukemia. Patients with ALL have presented with shorter telomeres than those with AML. In contrast, the telomeres of AML patients become shorter at a faster rate than those of ALL patients when the disease worsens [186,189]. The shortest telomeres are usually found in ALL and AML cells with cytogenetic abnormalities, while the lowest levels of telomerase expression and longer telomeres are found in AML [186,189].

Significant differences have been observed between ALL and AML cells regarding telomerase expression. Initially, telomerase is higher at the time of diagnosis or progression of AML patients than at the time of remission in the disease, and it corresponds with hTERT but not with an RNA template (hTR) expression in AML [192]. In line with this, AML patients with several cytogenetic abnormalities have presented with the shortest telomeres [193].

In adults, telomerase expression seems to be highest in B-cell ALL, followed by AML and T-cell ALL [186]. An increase in telomerase activity is not simply related to an overall rise in TERT expression but to altered levels of expression of the different isoforms of TERT [186]. Consistent with this, telomere length values were also observed to be higher in AML, and the telomere length values followed a decreasing trend first in T-cell ALL and then in B-cell ALL, confirming the reversing action of telomerase against telomere shortening [186]. Meanwhile, AML and B-cell ALL presented higher hTERT and telomerase expression, followed by T-cell ALL in children [194].

In addition, the prognostic value of telomere length in AML is substantiated since the overall survival of AML patients is prolonged for six months when their telomeres are elongated [195]. In particular, longer telomere length values are proven to be associated with mutations in the epigenetic modifying enzymes, the IDH1/2 genes; a positive relationship has been observed between IDH mutations and positive clinical outcomes in AML [196]. This study provides initial evidence about the positive prognostic role of telomere length elongation on the overall survival of AML patients [195]. Consistent with this, it was also revealed that bone marrow mononuclear cells from patients with acute myeloid leukemia presented an accelerated telomere shortening rate from their diagnosis to their relapse. On the contrary, telomere length elongation was observed in the bone marrow mononuclear cells of diagnosed patients during their chemotherapy-induced remission [197]. In a recent study, thirteen telomere-regulated genes (TRGs) were evaluated for the prognosis of AML [198]. In particular, B cells, T helper cells, natural killer cells, tumor-infiltrating lymphocytes, regulatory T (Treg) cells, M2 macrophages, neutrophils, and monocytes were more prevalent in the group of AML patients with a high expression of the TRG signature, supporting its prognostic value for poorer overall survival among AML patients [198]. Accordingly, the TRG signature can aid in risk assessment, thus guiding specialized treatment approaches [198]. As a result, a potential predictive and therapeutic biomarker can be generated by combining the characteristics of the immune cell landscape and the expression of telomere-related genes. Interestingly, approximately 10% of patients with TBDs are at high risk of developing cancer, primarily myeloid neoplasms (MNs) such as myelodysplastic syndrome (MDS) and acute myeloid leukemia (AML) [199]. Notably, patients with TBD are more likely to develop clonal hematopoiesis (CH), which is characterized by a clonal growth of mutations in myeloid genes in hematopoietic stem and progenitor cells, preceding the occurrence of MDS/AML [200]. Dyskeratosis Congenita is the most prevalent TBD. The detection of a disease-causing mutation in one of the genes influencing telomere length balance, specifically DKC1, TERC, and TERT, can contribute to the identification of TBD [201].

In this context, patients with Hodgkin’s lymphoma (HL) and non-Hodgkin’s lymphoma (NHL) who underwent autologous hematopoietic stem cell transplantation (aHCT) one to three years prior experienced recurrence, developing acute myeloid leukemia (AML) or therapy-related myelodysplasia (t-MDS) due to reduced telomere length values [202]. After autologous transplantation for lymphoma, therapy-related myelodysplasia or acute myeloid leukemia may occur due to a persistent rate of telomere shortening in myeloid cells, regardless of other risk factors for these diseases, as evidenced by multivariate analysis [202]. One year following aHCT, short telomere length values in the hematopoietic cells of HL/NHL patients typically precede the onset of AML/t-MDS, suggesting the prognostic significance of telomere shortening in these diseases [202]. This accelerated telomere shortening observed in HL/NHL patients who relapse after aHCT likely reflects the rapid proliferation of the leukemic cell clone, along with the inactivation of telomerase during the early stages of leukemia [202]. Regarding myelodysplastic syndrome (MDS), the aforementioned results can be supported since a subgroup of patients with greater severity of MDS and complex karyotypic alterations show reduced telomere length reduction [203,204]. Some individuals with bone marrow (BM) failure syndromes have mutations in telomere-related genes [205]. For example, Dyskeratosis Congenita, an inherited BM failure disease, is linked to an increased risk of AML due to telomerase dysfunction [206].

Last but not least is a telomeric repeat-containing RNA (TERRA), which is transcribed from subtelomeric promoters toward chromosome ends. MLL-rearranged ALL and AML present the overexpression of TERRA [207,208]. Increased TERRA levels may be a potential marker, particularly in AML and MLLr-ALL, and they correlate with leukemic load [207,208].

To sum up, the aforementioned evidence supports the prognostic role of telomere length in ALL survival and aggressiveness. In the panel of prognostic makers in ALL disease, telomerase also has its value. Similar findings have been proven in AML pathogenesis.

## 7. Telomerase—A Potential Therapeutic Target in Leukemias

The primary function of telomerase is to preserve telomere length and compensate for the gradual loss of telomeres after replication cycles. Telomerase is a ribonucleoprotein complex comprising a telomerase RNA component (hTR), providing an RNA template and a catalytic protein with telomere-specific reverse transcriptase activity (hTERT), which extends hexameric TTAGGG repeats to the chromosomal ends [209]. The expression of hTERT has been associated with telomerase activity [209].

The vast majority of post-mitotic cells are senescent and are characterized by telomere shortening due to the lack of telomerase activity. On the contrary, the progenitor and cancer cells display high telomerase activity, contributing to their telomere length maintenance and unrestricted cell proliferation [63,210,211]. Numerous investigations discovered a connection between telomerase activity and the clinical aggressiveness of some cancers, such as leukemia [203].

Hematologic and solid tumors in adults and children are described to have short telomeres and detectable amounts of telomerase [212]. In adults, telomerase could provide prognostic information in CLL, myeloproliferative disorders, and solid malignancies [212,213]. In pediatric patients, telomerase has been described as a prognostic marker in both AML and ALL [214,215] and, for this reason, telomerase inhibitors are administered.

Telomerase is also an appealing target for cancer therapy. The enzyme is found in most analyzed cancer cells but is absent in nearly all normal somatic cells, making telomerase inhibitors highly specific and telomerase a universal oncology target. Furthermore, because normal cells have longer telomeres compared to cancer cells, the toxicity of these inhibitors in normal tissues is minimal [216,217].

**Figure 3:**
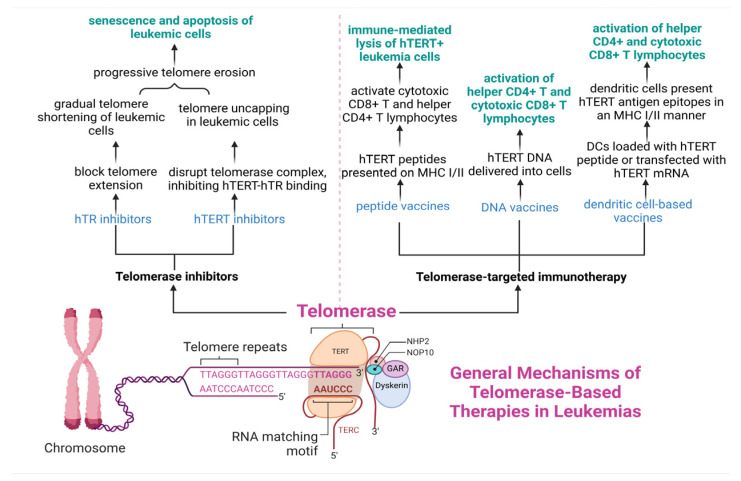
The telomerase-based therapeutic options in leukemias. Telomerase inhibitors include hTR inhibitors that block telomere extension and hTERT inhibitors that disrupt telomerase complex formation, leading to telomere shortening or uncapping and triggering senescence or apoptosis of leukemic cells. In telomerase-targeted immunotherapy, peptide vaccines present hTERT antigens via major histocompatibility complex (MHC) I/II to activate CD8^+^ and CD4^+^ T cells. DNA vaccines deliver hTERT DNA to induce effector T cell responses. In dendritic cell-based vaccines, dendritic cells are loaded with hTERT peptides or transfected with hTERT mRNA, and they present hTERT epitopes to stimulate cytotoxic CD8^+^ and helper CD4^+^ T cells for killing leukemia cells (created with BioRender.com).

Firstly, it is essential to distinguish between telomerase-based therapies; on the one hand, therapeutic approaches based on telomerase inhibition are designed to directly inhibit the enzyme, thus hindering its telomere maintenance mechanism. This leads to the progressive shortening of telomeres, induced senescence, and culminates in apoptosis [218]. Telomerase inhibitors may trigger both a delayed antiproliferative response via a gradual mechanism, mostly involving telomere degradation (e.g., hTR inhibitors) or an immediate antiproliferative response through a rapid mechanism, primarily centered on telomere uncapping (e.g., hTERT inhibitors) (Figure 3) [219].

On the other hand, a vaccination containing antigen-presenting cells that have been either exposed to high concentrations of an immunogenic hTERT peptide or engineered to overexpress an immunogenic portion of hTERT is used in telomerase-targeted immunotherapy (Figure 3). After being administered to a patient, antigen-presenting cells trigger the production of a specific immune response against the telomerase antigen epitope. These cells can identify and eliminate cancer cells that express telomerase (Figure 3) [220]. Telomerase vaccines can be DNA, peptide, or dendritic cell-based vaccines that trigger tailored and powerful anti-leukemia immune responses (Figure 3).

Considerable research has focused on developing drugs targeting telomerase for cancer treatment. Despite the ongoing challenges, strategies aimed at telomerase hold significant potential for revolutionizing cancer therapy. Recent advancements in computational models of human telomerase enhance the likelihood of developing clinically effective drugs [221].

### 7.1. Telomerase Inhibitors in Leukemias

Telomerase inhibitors can restrict the growth of malignancies and cause gradual telomere shortening by inducing the DNA damage response, thereby causing senescence or apoptosis [222,223]. In addition, telomerase activity plays a crucial role in maintaining leukemia stem cells’ ability to self-renew and telomere length values. In this regard, telomerase suppression can interfere with LSC efficacy, thus compromising the risk of leukemia recurrence [224].

In the field of telomerase inhibitors, a recently approved drug, imetelstat, offers promising results. The molecular mechanism of imetelstat is based on interfering with telomerase’s association with the catalytic subunit by attaching to the intrinsic RNA template with considerable affinity and specificity [225]. Imetelstat is an hTR inhibitor that acts by interfering with the RNA component of telomerase (hTR) (Figure 3). This competitive telomerase inhibitor received FDA approval in 2024 for treatment [226]. Recent preclinical and clinical studies have shown the effectiveness of imetelstat in hematologic malignancies, specifically myeloproliferative neoplasms, myelodysplastic syndromes, and AML [212,227]. The imetelstat is considered FDA-approved for treating myelodysplastic syndromes (MDS), and is being studied for myelofibrosis (MF) and acute myeloid leukemia (AML) [228]. In AML, imetelstat selectively diminishes leukemia stem cells (LSCs), which, when paired with chemotherapy, delays the disease course and lowers the potential for recurrence [229]. In line with this, the clinical benefit of imetelstat is seen in the case of myeloproliferative neoplasms (MPN) that can convert into cases of AML. In MPN patients treated with the telomerase inhibitor GRN163L (imetelstat), two phase II clinical trials have been conducted, demonstrating a positive effect of imetelstat as patients responded positively [230]. However, telomerase inhibition does not constitute the main mechanism of action of imetelstat in MPN, as some of the clinical responses of patients are considered the result of the medication’s adverse effects, such immunosuppression [231]. Imetelstat treatment has demonstrated its clinical benefit in patients with essential thrombocythemia, characterized by an excess of platelets, who did not respond to previous medications or experienced intolerable side effects [232].

In addition, imetelstat and dasatinib efficiently reduce TKI-resistant LSCs during the blast phase of chronic myeloid leukemia (CML) by suppressing β-catenin signaling cascade [224]. From a molecular perspective, the mechanism of imetelstat is based on reducing the telomerase levels and activity, as imetelstat is designed to target the RNA component of telomerase (Figure 3) [212,227]. Likewise, in patients with myelofibrosis who had relapsed or were unresponsive to a JAK inhibitor, imetelstat delayed fibrosis in 21% of patients [230].

The structure of the telomerase holoenzyme was poorly characterized until recently, which hindered the development of small-molecule inhibitors. BIBR1532 is a small-molecule hTERT inhibitor that reduces telomerase activity, ultimately leading to telomere uncapping. BIBR1532 induces telomere shortening in numerous solid tumor cell lines in vitro and triggers p53-mediated apoptosis in an acute myeloid leukemia cell line, but not in nonmalignant hematopoietic cells (Figure 3). Additionally, BIBR1532 inhibited hTERT expression in primary chronic myelogenous leukemia cells and pre-B-cell acute lymphoblastic leukemia cells [223,229,233]. As a result, BIBR1532 seems to cause apoptosis and telomere shortening in AML and CLL cells in a p53-dependent manner. However, the clinical development of BIBR1532 has been hampered by its poor pharmacokinetic and bioavailability characteristics [229].

In this context, Li et al. discovered a compound, IX, an imatinib derivative with a telomerase inhibitor fragment substitution, which eliminated LSCs without affecting HSC survival [234]. It reduces drug-resistant K562/G and blast crisis CML primary patient cell viability [234] (Figure 3). Additionally, IX can lower LSC frequency, colony-forming abilities, and survival. In vivo, IX reduced tumor burden in the PDX model and prolonged lifetime [234]. Compound IX reduces telomerase activity and alternative telomere lengthening. IX also inhibits canonical and non-canonical Wnt pathways [234]. This new molecule offers promising perspectives in the treatment of drug-resistant leukemia, since its molecular mechanisms of action rely on combinatorial targeting of signaling pathways and telomerase action (Figure 3) [234].

Telomerase can also be used as a therapeutic option for ALL in the face of effective telomerase suppression, particularly in individuals with a high-risk profile [214]. According to the results of a recent study, imetelstat seems to exert a dose-dependent suppressive effect on primary lymphoblasts of high-risk acute ALL children, since high-risk pediatric ALL patients presented increased telomerase levels compared to those of non-high-risk patients [214]. The elevated telomerase in high-risk pediatric ALL patients could be the cause of higher telomere length values in lymphoblasts and B and T lymphocytes of ALL children compared to those of ALL adults [214].

Last but not least, telomerase inhibitors have demonstrated increased potency when used with conventional chemotherapeutic drugs, which may help overcome the resistance of leukemia cells to standard chemotherapeutic options and improve treatment results [235,236].

Despite significant progress, no telomerase inhibitors have gained approval for leukemia treatment, underscoring the need for ongoing research to identify new compounds with enhanced specificity and efficacy. Large-scale clinical trials are essential to assess the long-term efficacy and safety of these therapies across various leukemia subtypes. Current findings suggest that telomerase inhibitors exhibit low toxicity in normal cells, primarily affecting malignancies associated with high telomerase activity and short telomeres.

### 7.2. Telomerase-Based Immunotherapy in Leukemias

Towards developing leukemia treatment, the emergence of telomerase immunotherapy provides novel insights into the potential therapeutic options against leukemias, leveraging telomerase and the host immune system. Telomerase-based immunotherapy has garnered considerable interest in recent years. Two methods of effective telomerase immunotherapy have been described: human telomerase reverse transcriptase (hTERT) peptide administration and the use of dendritic cell-based telomerase vaccines [237]. In both cases, the cancer cells are killed explicitly by inducing the host’s cell immunity in an hTERT-specific manner. Apart from these, TERT peptide and dendritic cell-based approaches, cell and DNA-based approaches exist. In cell-based approaches, dendritic cells were transfected with an adenovirus containing TERT cDNA. These cell-based TERT approaches have been examined in both in vitro and in vivo settings [238,239].

In the field of telomerase peptide vaccines, Vx-001, UV1, UCP-Vax, and GV1001, are regarded as essential peptide vaccines that elicit hTERT-specific immune responses [240,241]. The Vx-001 includes two hTERT peptides that are presented on the MHC I complexes of antigen-presenting cells, causing the killing of cholangiocarcinoma, non-small cell lung cancer, melanoma, and breast cancer cells by cytotoxic CD8^+^ T lymphocytes [242]. In addition to this, the Vx-001 telomerase peptide vaccine is effective in low immunogenic tumors with low penetration of tumor-infiltrating lymphocytes [243]. The UV1 peptide vaccine involves three hTERT peptides and has been examined with checkpoint inhibitors. When patients with malignant mesothelioma were treated with ipilimumab (cytotoxic T lymphocyte-associated antigen-4, CTLA-4 inhibitor) and nivolumab (programmed death 1 blocker, PD-1 inhibitor) in combination with UV1, they recovered more rapidly, eliciting immediate immune responses [244]. Likewise, further 5-year monitoring of metastatic melanoma patients receiving UV1 in conjunction with ipilimumab revealed the accumulation of UV1-specific helper CD4^+^ T lymphocytes [245]. Accordingly, UCP-Vax consists of four universal cancer peptides that map hTERT, and they are presented in a major histocompatibility complex (MHC) class II manner, showing good tolerance and significant clinical efficacy against non-small lung cancer, hepatocellular carcinoma, glioblastoma, and human papillomavirus-associated tumors [246].

Several clinical trials have provided clues that the GV1001 is an approved hTERT peptide vaccine that is effective in hepatocellular carcinoma, pancreatic cancer, non-small cell lung cancer, prostate cancer, and melanoma [247,248,249,250]. Interestingly, a clinical trial has shown that GV1001 (mapping the 611–626 amino acid sequence of hTERT) and HR2822 (mapping the 540–548 amino acid sequence of hTERT) are telomerase peptide vaccines that exert effective cytotoxic immune responses in an HLA-A2 restricted manner, thus contributing to the increased clinical response of patients with non-small lung cancer [249]. In addition, the GV1001 peptide vaccine has been reported to ameliorate T-cell responses in patients with advanced lung and pancreatic cancer in phase I/II trials, without causing any clinical adverse effects [251]. The privilege of this GV1001 telomerase peptide vaccine is based on stimulating cytotoxic CD8^+^ T lymphocytes and helper CD4^+^ T lymphocytes, regardless of patients’ HLA typing [252,253].

Overall, the telomerase peptide vaccines are promising for combatting malignancies due to their ease of manufacture and high specificity, which itself is due to their high affinity with antigens. Despite the above, there are several challenges that should be addressed to enhance their clinical efficacy. First of all, telomerase peptide vaccines induce specific immune responses. The peptides are presented in an MHC-restricted manner by antigen-presenting cells. Secondly, they are characterized by low immunogenicity, which is improved with the help of adjuvants [240].

**Figure 4:**
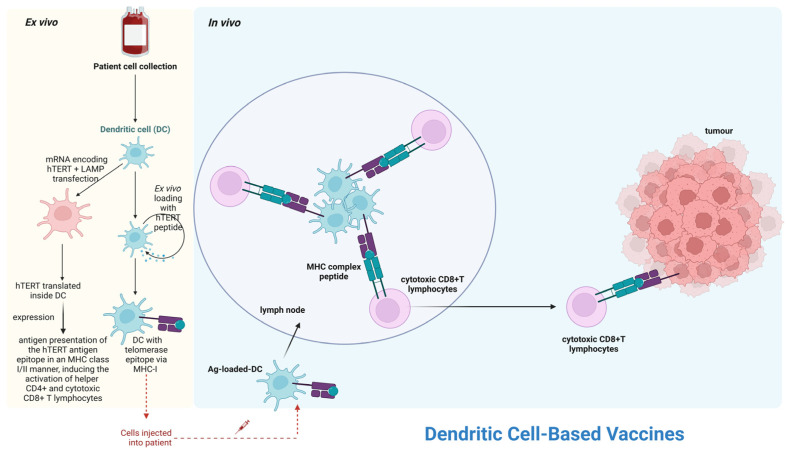
The molecular mechanisms underlying dendritic cell-based vaccines. This figure illustrates the mechanism of dendritic cell (DC)-based telomerase vaccines. Patient-derived DCs are either transfected with mRNA encoding human telomerase reverse transcriptase (hTERT) and lysosomal-associated membrane protein 1 (LAMP) or ex vivo loaded with hTERT peptide. Transfected DCs translate and present the telomerase epitope via major histocompatibility complex (MHC)-I and (MHC)-II, while peptide-loaded DCs present it via MHC-I. After injection into the patient, antigen-loaded DCs migrate to lymph nodes and activate cytotoxic CD8^+^ T lymphocytes, recognizing and killing hTERT-expressing tumor cells (created with BioRender.com).

In the field of dendritic-based cancer vaccines, dendritic cells have been employed in cancer immunotherapy over the past ten years to elicit strong antitumor immune responses [254]. Dendritic-based cancer vaccines offer significant advantages since they stimulate cytotoxic CD8^+^ T lymphocytes via MHC class I, generating strong and robust immune responses [255]. In particular, activated CD8^+^ T cells multiply and convert into cytotoxic T lymphocytes, which eventually migrate to the tumor region to eradicate numerous cancer cells [255]. To eliminate cancer cells, cytotoxic T lymphocytes secrete pro-inflammatory mediators, including cytokines, perforin, and granzyme [255]. An hTERT tumor-associated antigen is included as a central part of research into this dendritic cell-based immunotherapy option against cancer (Figure 4) [256]. Interestingly, dendritic cell-based telomerase vaccines can be formed in two different ways. On one side, the in vitro activation of dendritic cells with hTERT antigen epitope and the infusion of stimulated antigen-presenting cells into the host ex vivo boost the immune system to eliminate tumor cells [257]. On the other side, dendritic cells fulfill their antigen-presenting role through the overexpression of hTERT antigen epitope (Figure 4) [258].

Regarding the dendritic cell-based telomerase vaccine, p540 (peptide sequence—ILAKFLHWL) was revealed as the first tumor-associated antigen (TAA) peptide as a part of hTERT, presented by the surface of cancer cells in a human leukocyte antigen (HLA) class I manner (Figure 4) [259]. These telomerase antigen epitopes presented on the HLA class I surface of cancer cells can cause the activation of cytotoxic T lymphocytes to eliminate hTERT^+^ tumors from several histologic origins (Figure 4) [260]. In addition, potent cytotoxicity by cytotoxic CD8^+^ T lymphocytes has been demonstrated against the following tumors, including carcinoma, sarcoma, myeloma, and melanoma, as well as newly isolated lymphoma and leukemia cells, in an HLA-A*0201-specific manner (Figure 4). In addition to the lysis of leukemia cells by cytotoxic T lymphocytes, the hTERT epitope can be recognized by antigen-presenting cells, thereby boosting either the promotion of helper CD4^+^ T cells or cytotoxic CD8^+^ T cell responses (Figure 4) [241,261].

In B-cell chronic lymphocytic leukemia (B-CLL), natural antileukemic cytotoxic T cells targeting telomerase-derived antigens are generated [262]. In particular, dendritic cells loaded with the hTERT epitope cause the increased formation of hTERT-specific cytotoxic CD8^+^ T lymphocytes (Figure 4) [262]. In this way, the concept of a telomerase vaccination strategy that utilizes cytotoxic T cells to target tumor cells expressing telomerase is supported (Figure 4) [35].

Despite the advances in AML therapy, relapse rates remain high and AML’s overall survival is inferior to ALL. For this reason, an autologous telomerase-based dendritic cell vaccination called AST-VAC1 (formerly called GRNVAC1) has effectively completed a phase 2 clinical trial in ultimately relapsed patients with acute myeloid leukemia (AML) (Figure 4) [35]. Patients received mature autologous dendritic cells loaded ex vivo with mRNA encoding telomerase and a part of lysosomal-associated membrane protein 1 (LAMP), ensuring the degradation of TERT into small peptides (Figure 4) [258]. In this way, dendritic cells present the telomerase antigen epitopes in a MHC class I or II to CD8^+^ T or helper CD4^+^ T lymphocytes, boosting durable and specific immune responses against leukemia cells (Figure 4) [237,238,263,264]. On one side, the hTERT antigen of tumor cells forms a complex with an MHC class I of antigen-presenting cells, which activate CD8^+^ T cells and induce their transformation into cytotoxic T cells that attack cancer cells through increased secretion of the following factors: perforin, cytokines, and granzyme (Figure 4) [242]. On the other side, the antigen-presenting cells phagocytose the tumor antigens supplied by cancer cells after they have succumbed (Figure 4) [242]. The released hTERT antigenic peptides are presented on MHC class II of antigen-presenting cells, activating CD4^+^T cells to assist the killing capacity of cytotoxic CD8^+^ T lymphocytes (Figure 4) [242]. The activated CD4^+^ T cells undergo differentiation into helper T lymphocytes that mediate their action through increased release of pro-inflammatory cytokines (Figure 4) [242]. In this way, the tumor immune evasion is thwarted to encourage a long-lasting anti-cancer response (Figure 4) [265]. In addition, the increased synthesis of antibodies in the fight against cancer cells is induced after the stimulation of helper T lymphocytes by the hTERT antigen peptide-MHC class II complexes at the surface of antigen-presenting cells (Figure 4) [242].

In the case of metastatic prostate cancer, the cytotoxic and helper immune responses are generated to a greater extent in patients who have received dendritic cells transduced with mRNA encoding hTERT than in patients without a dendritic cell-based vaccine [264]. In line with this, advanced prostate or breast cancer patients have shown cancer remission through the induction of cytotoxic T cell response after the vaccination of patients with dendritic cells loaded with hTERT 1540 epitope [266]. Other clinical trials, phase I or II, have illustrated that dendritic cells loaded with TERT p540 peptide sustain the clinical response of prostate, breast, lung cancer, and melanoma patients [257]. Accordingly, dendritic cells loaded with hTERT p865 peptide (RLVDDFLLV amino acid sequence) have also shown increased clinical efficiency in combating tumor burden [267]. In this study, fibroblast-derived artificial antigen-presenting cells loaded with either TERT p540 or hTERT p865 peptide were generated to activate cytotoxic CD8^+^ T lymphocytes in an HLA class I A*0201 manner to avoid using antigen-presenting cells on an autologous basis [267]. Overall, the intensity of cytotoxicity against hematologic and epithelial malignancies is associated with the degree of hTERT activity in different malignancies [267].

In the clinical setting, GRNVAC1 is effective and well-tolerated by patients, without apparent autoimmune responses [268]. Consistent with this, another study provides compelling evidence that engineering dendritic cells with hTERT antigen can mediate the lysis of AML cells through the activation of the immune system in a good, tolerable, and safe manner [269]. The prolonged administration of GRNVAC1 is linked to ameliorating disease-free survival in high-risk AML patients through the increased generation of cytotoxic CD8^+^ T lymphocytes against the immunogenic hTERT epitope loaded by dendritic cells [269]. Like GRNVAC1, GRNVAC2 is another dendritic cell-based vaccination option produced using human embryonic stem cells rather than leukapheresis. Regarding the delivery system, GRNVAC2 is considered more appropriate than the GRNVAC1 vaccine. Interestingly, these dendritic cell-based cancer vaccines are regarded as very effective against cancers with unknown T-cell epitopes due to their lack of human leukocyte antigen (HLA) restriction [251,270].

Last but not least, in another clinical trial, the clinical efficacy of the telomerase-based dendritic cell vaccine has been illustrated in advanced pancreatic cancer [271]. In particular, the hTERT antigen epitope of pancreatic cancer cells, in combination with survivin (SRV.A2) and carcinoembryonic antigen (CEA), can bind to the HLA-A2 on the surface of dendritic cells, forming the antigen peptide-MHC class II complexes in antigen-presenting cells, which in turn trigger cytotoxic CD8^+^ T lymphocytes that specifically target pancreatic cancer cells in an hTERT-dependent manner [271]. As a result, this telomerase-based dendritic cell vaccine increases the formation of hTERT-specific T cells, thus preserving the clinical picture of pancreatic cancer patients and preventing the potential of metastasis [271].

In addition, hTERT DNA vaccines can also boost strong cytotoxic CD8^+^ or helper CD4^+^ immune responses, including the heightened expression of pro-inflammatory molecules, such as tumor necrosis factor-α (TNF-α), interferon-gamma (IFN-γ), [272,273]. In more detail, the DNA encoding the inactivated hTERT form is transferred into cancer cells through electroporation, thus causing the presentation of the hTERT antigen epitope to antigen-presenting cells and the subsequent activation of either cytotoxic or helper T immune response. Interestingly, the hTERT DNA approach is superior to the hTERT approach owing to the low cost and potential for infection [274]. The main beneficial features of DNA vaccines are focused on their long-term reliability, assurance, and ease of manufacture. Despite these advantages mediated by DNA vaccines, the immune responses are attenuated, mainly when the antigens are internal proteins and due to the dearth of a suitable delivery system of DNA vaccines, significantly compromising their immunogenicity [275,276].

In the field of dendritic cell-based vaccines, the hTERT-targeted vaccines provide deep insights into immunotherapy since they have the following advantages: (1) ease and comfortability, (2) simplicity and reliability, (3) cost-effectiveness and easy manufacturing, (4) high specificity and efficacy due to high affinity of telomerase antigen peptide to T cell receptor (TCR) of T cells, and (5) minimal risk for undesirable effects [242]. However, numerous obstacles can be overcome to enhance the hTERT-targeted specificity and efficacy while lowering the possible autoimmune effects.

To sum up, telomerase-based vaccines can mediate different immune responses due to the patient’s immunity and tumor microenvironment, contributing to the heterogeneity of the clinical effectiveness mediated by telomerase-based vaccines. The need for ongoing research for all immunotherapeutic telomerase-based options is urgently needed, particularly in combination with other immunotherapy strategies like checkpoint inhibitors.

## 8. Conclusions

In conclusion, telomere dysfunction significantly influences clinical outcomes in the four primary types of leukemia. This review examines the intricate relationship between leukemia, telomere length, and telomerase activity. The essential role of telomeres and telomerase highlights their prognostic importance, suggesting potential value in the clinical management of leukemia patients. Increasing evidence advocates for the routine telomere length assessment as part of prognostic evaluation in CLL, CML, AML, and ALL, especially when combined with established prognostic indicators. Moreover, leukemias are characterized by elevated levels of oxidative stress and mitochondrial dysfunction, ultimately resulting in telomere shortening and genomic instability. In light of the molecular mechanisms behind telomere dysfunction, telomerase-targeted immunotherapeutic strategies are being explored in an unprecedented manner, reflecting the urgent need to elicit specific and robust immune responses that effectively limit tumor cells expressing telomerase epitopes.
